# Chemotherapy in conjunction with traditional Chinese medicine for survival of patients with early female breast cancer: protocol for a non-randomized, single center prospective cohort study

**DOI:** 10.1186/s13063-019-3848-8

**Published:** 2019-12-17

**Authors:** Chien-Ting Liu, Yen-Hao Chen, Yu-Chuen Huang, Shih-Yu Chen, Ming-Yen Tsai

**Affiliations:** 1 Division of Oncology, Department of Internal Medicine, Kaohsiung Chang Gung Memorial Hospital and Chang Gung University College of Medicine, Kaohsiung, 83301 Taiwan; 2 Department of Medical Research, China Medical University Hospital and School of Chinese Medicine, China Medical University, Taichung, 41354 Taiwan; 3 Department of Pediatrics, E-Da hospital and I-Shou University, Kaohsiung, Taiwan; 4 Department of Chinese Medicine, Chang Gung Memorial Hospital - Kaohsiung Medical Center, Chang Gung University College of Medicine, Kaohsiung, 83301 Taiwan

**Keywords:** Traditional Chinese medicine, Quality of life, Study design, Disease-free survival, Breast cancer

## Abstract

**Background:**

Chemotherapy after surgery for breast cancer plays a fundamental role in reducing the risk of distant and local recurrence. An increasing number of patients seek traditional Chinese medicine (TCM) during adjuvant chemotherapy to relieve symptom discomfort and side effects as well as to strengthen the body’s defenses. However, evidence on how concurrent TCM treatment affects prognosis is scarce. This trial aims to evaluate the association between TCM treatment and disease-free survival outcomes for patients with early breast cancer who are undergoing adjuvant chemotherapy.

**Methods/design:**

This is a non-randomized, single center, prospective cohort study begun in November 2018 in Kaohsiung, Taiwan. A sample of 310 participants diagnosed with early breast cancer was recruited from the Breast Cancer Research Team and will be followed up every 3 to 6 months until October 2023. Detailed information of the participants, including general information, history of cancer, quality of life, side effects and safety of treatment, TCM body constitution, and meridian energy analysis, was collected face to face at baseline.

**Discussion:**

This is the first prospective observational cohort study on TCM in patients with early breast cancer who are receiving adjuvant chemotherapy to evaluate the prognosis. Through this trial, we hope to assess the feasibility of a larger-scale clinical trial in the future and formulate an integrated TCM care program.

**Trial registration:**

ClinicalTrials.gov, NCT03797248. Registered on 5 January 2019.

## Background

Breast cancer is the most common cancer in women, accounting for 25.1% of all cancers worldwide [1]. Breast cancer has been the fastest-growing incidence of cancer in Taiwan over the past 10 years, with a growth rate approaching 58% and an incidence of 188–194 per 100,000 women-years [2].

Chemotherapy plays an important role in the systemic treatment of breast cancer, and it is the cornerstone of therapy for patients who are not candidates for hormone therapy [3]. An Early Breast Cancer Trialists’ Collaborative Group (EBCTCG) meta-analysis study reported that taxane-based or anthracycline-based chemotherapy may decrease cancer recurrence and mortality, largely independent of age, lymph node metastasis, or estrogen receptor status [4]. Currently, after breast surgery, National Comprehensive Cancer Network (NCCN) guidelines specify only adjuvant chemotherapy to eliminate remaining cancer cells and improve survival in most patients with early stage breast cancer [5, 6]. Besides killing cancer cells, chemotherapeutic agents can also damage healthy tissues, leading to side effects that negatively affect patients’ quality of life (QOL) and compliance with cancer treatment [7]. Therefore, there is a clinical need to find an intervention to manage the discomfort of chemotherapy and improve patients’ tolerance and well-being.

Increasing numbers of patients use a wide range of complementary and alternative medicine (CAM) therapies, including herbs, vitamins, homeopathic remedies, and Chinese herbal medicines (CHMs), during their anticancer treatment. The use of CHMs based on traditional Chinese medicine (TCM) theory for breast cancer has been recorded in ancient Chinese books for more than 2000 years. In Taiwan, nearly 20% of patients seek CHM as part of the treatment for their breast cancer [8]. As a CAM therapy for breast cancer, CHM has gradually shown its effects in controlling the progression, increasing the susceptibility to radiotherapy and chemotherapy, elevating immunity, and decreasing the toxicities or side effects of cancer therapies [9]. Authors of a systematic review demonstrated that the combined use of CHM with chemotherapy was an appropriate adjunctive therapy for immediate tumor response and chemotherapy-induced toxicities [10], but the supporting evidence was insufficient because most of the randomized controlled trials (RCTs) had poor study design. In addition, it is unproven whether combined CHM therapy is more effective than the standard adjuvant chemotherapy for clinical prognosis, especially in early breast cancer. Therefore, we planned to perform a single center prospective cohort trial to investigate whether the intervention of CHM can improve the prognosis of postoperative breast cancer patients in terms of disease-free survival (DFS), and then to provide evidence that TCM treatment is effective and safe. Meanwhile, this study can provide a breast cancer patient information database, which may correct the guidelines for TCM treatment, build trust between Western medicine doctors and TCM practitioners, and enhance the role of TCM in integrated medical care.

## Methods/design

### Study design

This study is a non-randomized, single center, prospective cohort study. The study began in November 2018 and is currently ongoing. A cohort of 310 patients aged more than 20 years with breast cancer with histologically diagnosed stages 1–3 after partial or radical surgery were enrolled from an academic medical center. All participants will complete six to eight cycles of adjuvant chemotherapy, which may last 6 months. The standard chemotherapy regimens include anthracycline-, taxane-, or anthracycline plus taxane-based chemotherapy specified by the NCCN guidelines, based on their disease states. At the time the participants enter the study, the time between surgery and initial chemotherapy is not more than 1 month. The study consists of two stages. Stage I is a cross-sectional study at baseline, and stage II is a cohort study across 3 years of follow-up. Participants will be followed up every 3 to 6 months for 3 years, until recurrence or death. The feasibility and precision of the entire process will be overseen by two supervisors. To ensure that patients adhere to the follow-up schedule, we remind patients of their scheduled visits by telephone or text message. Additionally, incentives are used to express appreciation for the participants’ recruitment and retention throughout the years.

### Study settings and participants

The study is being performed in Kaohsiung Chang Gung Memorial Hospital (KCGMH) in Taiwan. Approximately 2–4 weeks after the completion of surgery, the early breast cancer patients are hospitalized for port-a-catheter implantation and administration of the first cycle of adjuvant chemotherapy. At that time, we are referred to the patients by the Breast Cancer Research Team on the day of their hospitalization. Participant screening is implemented by our department specialists with a background in oncology. For eligible patients, the researcher provides details on the purpose, specific content, instructions on how to complete the trial, and precautions of cancer treatment. After we obtain their informed consent, patients are enrolled in the study.

**Fig. 1:**
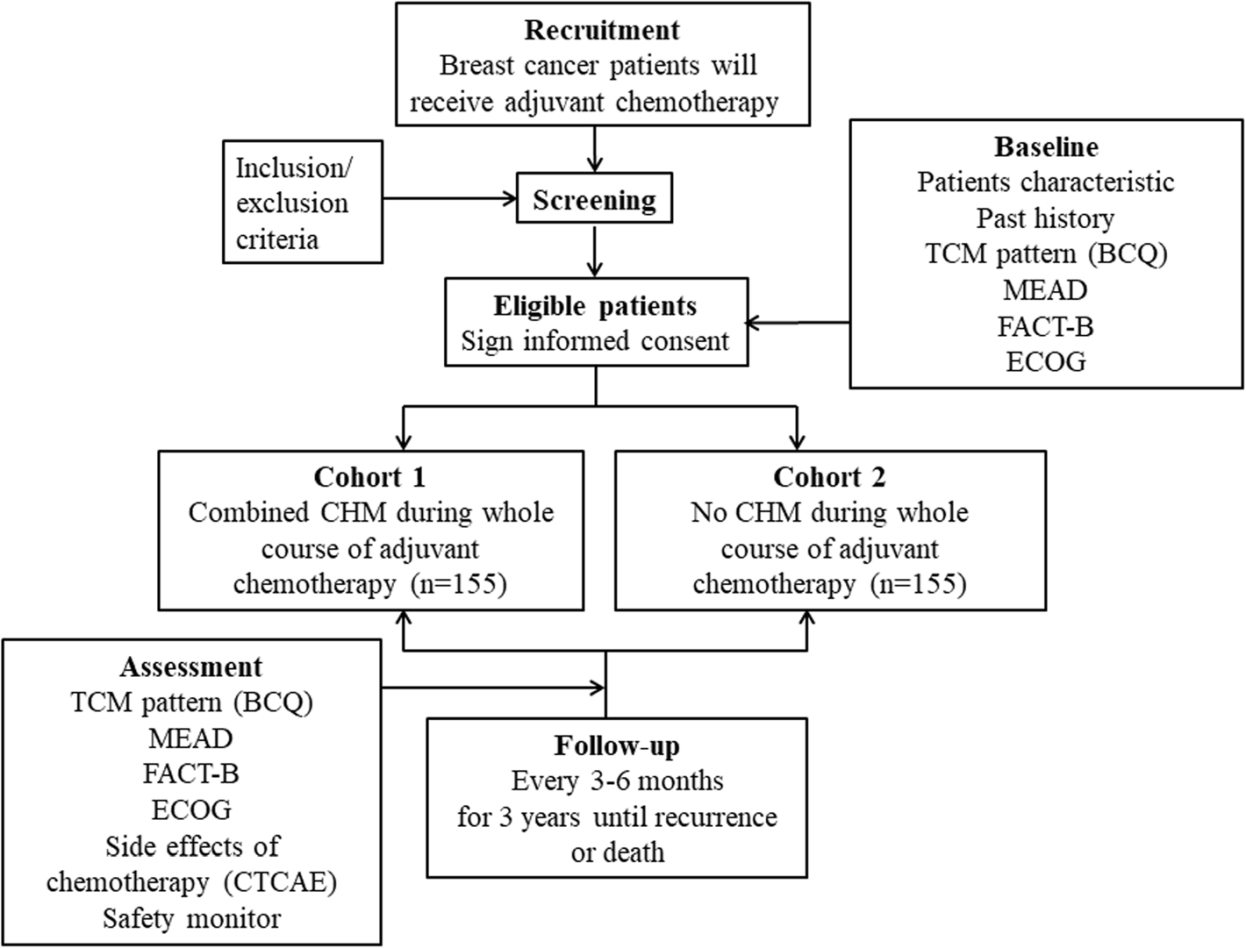
Flow chart of the study. The safety assessment contains a routine blood test, kidney function test, and liver function test. *BCQ* Body Constitution Questionnaire, *CHM* Chinese herbal medicine, *CTCAE* Common Terminology Criteria for Adverse Events (version 4), *ECOG* Eastern Cooperative Oncology Group (score), *FACT-B* Functional Assessment of Cancer Therapy - Breast Cancer, *MEAD* Meridian Energy Analysis Device, *TCM* traditional Chinese medicine

During the period of adjuvant chemotherapy, the participants can decide whether to accept the CHM intervention based on their personal conditions and evaluation of their own physiques. If a patient is willing to see TCM practitioners, we arrange CHM treatment for her. However, participants treated with CHM need to receive such treatment for at least half of the whole period. Participants taking CHM during the period of adjuvant chemotherapy are referred to as cohort 1, and patients receiving only chemotherapy without CHM belong to cohort 2. CHM is prescribed by TCM practitioners with more than 5 years of experience according to the TCM Treatment for Breast Cancer protocol developed in our Integrative Cancer Center (Table 1). The protocol and consensus assessment used in the study are based on the integration of empirical data and previous research of TCM syndromes in breast cancer [11, 12]. We record the number of TCM clinic visits, common CHM prescriptions, and days of taking the medicine, but we do not interfere with the treatment of TCM practitioners. The study project and estimated recruitment numbers are presented in Fig. 1.

**Table 1:** Syndrome differentiation-based CHM treatments for patients with breast cancer during adjuvant chemotherapy period

Pattern type	Symptoms	Treatment principle	Prescriptions (components)
肝鬱氣滯 Stagnation of liver-*qi*	Depressive mood and other irritability, feeling wired or having mood swings, distension in the upper abdomen, or a sense of constriction in the chest, poor vision, bitter taste, dry throat, vexation and irritability, and strong pulse	Relieving *qi* stagnancy in liver	Chai Hu Shu Gan Tang 柴胡疏肝湯 (*Pericarpium (Per.) Citri Reticulatae*, *Radix (Rx.) Bupleuri*, *Rhizoma (Rz.) Chuanxiong*, *Fructus (Fr.) Aurantii*, *Rx. Paeoniae Alba, fried Rx. Glycyrrhizae, Rz. Cyperi*) or Xiao Yao San 逍遙散 (*Rx. Bupleuri*, *Rx. Angelicae Sinensis*, *Rx. Paeoniae Alba*, *Rz. Atractylodis Macrocephalae*, *Poria*, *Rx. Glycyrrhizae Preparata*, *Herba (Hb.) Menthae Haplocalycis*, *Rz. Zingiberis Recens*)
脾虛痰濕 Spleen deficiency with phlegm dampness	Feeling heavy and tired, poor appetite, abdominal distension, diarrhea, nausea, vomiting, dyspepsia, a plump greasy tongue, and soft pulse	Tonifying the spleen and removing dampness	Liu Jun Zi Tang 六君子湯 (*Rx. Ginseng*, *Rz. Atractylodis Macrocephalae*, *Poria*, *Fried Rx. Glycyrrhizae*, *Per. Citri Reticulatae*, *Rz. Pinelliae Preparatum*) or Wei Ling Tang 胃苓湯 (*Rz. Alismatis*, *Poria*, *Polyporus*, *Rz. Atractylodis Macrocephalae*, *Ramulus (Ram.) Cinnamomi*, *Rz. Atractylodis*, *Cortex (Cx.) Magnoliae Officinalis*, *Per. Citri Reticulatae*, *Rx. Glycyrrhizae Preparata*, *Rz. Zingiberis Recens*, *Fr. Zizyphi Jujubae*)
肝腎陰虛 Deficiencies of liver- and kidney-yin	Feeling annoyingly hot in chest area and in palms of hands and feet, hypochondriacal pain, back pain, blurred vision, dizziness, tinnitus, night sweats, red tongue with scanty fur, thin and rapid pulse	Nourishing yin of liver and kidney	Liu Wei Di Huang Wan 六味地黃丸 (*Rx. Rehmanniae Preparata*, *Fr. Corni Shan*, *Rx. Dioscoreae*, *Poria*, *Cx. Moutan*, *Rz. Alismatis*) and Er Zhi Wan 二至丸 (*Fr. Ligustri Lucidi*, *Hb. Ecliptae*)
痰(瘀)熱夾雜 Stagnation of heat and phlegm (or stasis)	Hard, swelling, or painful lump in the breast, extreme heat, thirst, bitter taste, headache, dark red tongue with a yellow coat, slippery and rapid pulse	Cleaning heat and reducing toxicity, removing the sludge, and eliminating the swelling	Huang Lian Wen Dan Tang 黃連溫膽湯 (*Caulis Bambusae in Taeniam*, *Fr. Aurantii Immaturus*, *Rz. Pinelliae Preparatum*, *Per. Citri Reticulatae*, *Poria*, *Rx. Glycyrrhizae*, *Fr. Jujubae*, *Rz. Zingiberis Recens*, *Rz. Coptidis*) or Xue Fu Zhu Yu Tang 血府逐瘀湯 (*Semen (Sm.) Persicae*, *Flos Carthami*, *Rx. Angelicae Sinensis*, *Rz. Chuanxiong*, *Rx. Paeoniae Rubra*, *Rx. Cyathulae*, *Rx. Bupleuri*, *Rx. Platycodi*, *Fr. Aurantii*, *Rx. Rehmanniae*, *Rx. Glycyrrhizae*)
氣血兩虛 Deficiencies of *qi* and blood	Paleness, weakness, weak or breathy voice, emotional exhaustion, palpitation, insomnia, cramps, numbness, pale tongue, weak pulse	Invigorating *qi* and enriching the blood	Gui Pi Tang 歸脾湯 (*Rx. Ginseng*, *Rx. Astragali*, *Rz. Atractylodis Macrocephalae*, *Poria*, *Sm. Zizyphi Spinosae*, *Arillus Longan*, *Rx. Aucklandiae*, *fried Rx. Glycyrrhizae*, *Rx. Angelicae Sinensis*, *fried Rx. Polygalae*, *Rx. Zingiberis Recens*, *Fr. Jujubae*)

A participant will be discontinued from the study if she withdraws from chemotherapy, if a severe adverse event (AE) occurs, or if the researchers, for any safety reason, judge the participant’s continuation in the study to be inappropriate.

### Inclusion criteria

Subjects are eligible to participate if they are female and meet the following criteria: (1) age > 20 years; (2) histologically proven stage 1–3 breast cancer after surgery; (3) duration from the end of radical surgery to the beginning of the trial of less than 1 month; (4) Eastern Cooperative Oncology Group (ECOG) Performance score of 0–2 points; (5) agreement to participate in this study and sign informed consent.

### Exclusion criteria

Subjects will be excluded if they meet any of the following criteria: (1) comorbidity of inadequate heart, liver, kidney, or hematopoietic function or other serious diseases; (2) pregnancy or lactation; (3) history of mental illness; (4) distant metastasis and/or expected lifetime of less than 3 months; (5) have accepted external TCM intervention such as acupuncture, moxibustion, cupping, etc.; (6) treatment with other medicinal herbs outside our hospital.

### Outcome measurements

One investigator will be responsible for the follow-up of each participant at baseline and every 3 to 6 months. The schedule for data collection and follow-up by telephone or outpatient visits is shown in Table 2.

**Table 2:** Trial schedule of enrollment, interventions, and data assessments

Time point	Screening	Period of adjuvant chemotherapy			Follow-up, months			
	−4–0 week	1	3	6	9	12	…	36
Enrollment								
Informed consent	X							
Eligibility screen	X							
General assessment								
Demographic data	X							
Medical history	X							
Treatment situation	X							
TCM program								
CHM intervention		X	X	X				
TCM cohort only	X	X	X	X	X	X	X	X
Assessment								
FACT-B	X		X	X				
ECOG	X		X	X				
BCQ	X			X				
MEAD	X			X				
Imaging examination (CT, type B ultrasonography, bone scan)	X		X	X	X	X	X	X
Tumor markers (CEA & CA15-3)	X		X	X	X	X	X	X
Safety indicators	X	X	X	X	X	X	X	X
Chemotherapy-induced side effect, CTCAE		X	X	X	X	X	X	X
Cohort migration					X	X	X	X
Combined medication					X			

*ECOG* Eastern Cooperative Oncology Group, *FACT-B* Functional Assessment of Cancer Therapy-B, *BCQ* Body Constitution Questionnaire, *MEAD* Meridian Energy Analysis Device, *CT* Computed tomography, *CEA* Carcinoembryonic antigen, *CA15-3* Cancer antigen 15-3, *CTCAE* Common Terminology Criteria for Adverse Events, *CHM* Chinese herbal medicine, *TCM* Traditional Chinese medicine

### Primary outcome

The primary outcome of this study is 3-year DFS, defined as the period after curative treatment when no disease can be detected. This indicator will be calculated at the end of the study. The outcome assessment with regard to survival, local and regional relapse, and distant metastasis will be obtained by either review of the hospital records system or contact by mail or phone. The date of last evaluation will be recorded if patients have neither recurrence nor metastasis unless they reach censored data or there is loss of follow-up.

### Secondary outcomes

To elucidate the clinical impacts of QOL, TCM pattern, meridian energy, and side effects of chemotherapy for the use of CHM or not in breast cancer patients during adjuvant chemotherapy, we will evaluate the following secondary outcomes:

1. The QOL measures used in our trial for patients with breast cancer are the Functional Assessment of Cancer Therapy - Breast Cancer (FACT-B) scale and the ECOG criteria. The FACT-B scale is a specific form of a cancer treatment function evaluation system for QOL in patients with breast cancerdeveloped by Brady et al. in 1997 [13]. It consists of a general version (FACT-G) for measuring the QOL of cancer patients and a breast cancer subscale (BCS). The FACT-B consists of 36 entries and is divided into 5 sections: physical well-being, social/family well-being, emotional well-being, functional well-being, and the BCS. Each entry is set in a hierarchical setting (0–4) where higher scores indicate better quality of life. The ECOG score was published by Oken et al*.* in 1982 [14]. The standardized criteria are highly detailed and are used to evaluate the toxicity criteria, response criteria, and definitions of response by organ site involvement. It is scored on a scale of 0 to 5 points, where higher scores indicate worse physical conditions. QOL measurements will be assessed before, at 3 months, and at 6 months of adjuvant chemotherapy.
2. To evaluate the relationships of the TCM pattern, meridian energy (*qi*), and use of CHM during the course of adjuvant chemotherapy, we selected the Body Constitution Questionnaire (BCQ), developed by Professor Su, to classify patients’ TCM patterns [15]. The BCQ contains 44 items on the following three constitution types: Yang-*qi* deficiency (19 items), Yin-blood deficiency (19 items), and Phlegm-Stasis syndrome (16 items). Symptom frequency and intensity are reported on a 5-point Likert-type scale. Scores of ≥31, 30, and 27 are respectively used as the thresholds for the three individual constitution types. A higher score indicates a more pronounced constitution. If a score indicates two or three of the constitution types, it is classified as a mixed type. If a score indicates none of the three, then it is classified as the Gentleness type. The objective tool for measuring the meridian energy of patients with breast cancer is the Meridian Energy Analysis Device (MEAD) (ME100; Med-Pex Enterprises, Taichung, Taiwan). The MEAD can reflect the conditions of certain organs through analysis of the symmetrical modified Yuan points on the wrists and ankles and comparison of their mutual relations and changes in micro-electrical currents to represent the physiological and pathological phenomena of the relevant meridians [16]. The variations in electrical conductance can reveal significant characteristics such as deficiency/excess or yin/yang related to TCM concepts. Therefore, it has been shown in previous studies to provide a reference for disease prognosis, treatment response, and even balance of the autonomic nerve system [17, 18]. The measurements are started with very low current and gradually increased to a maximum value of 200 μA. Recordings of the electrical conductivity of the meridians are entered directly into a computerized system. The conductivity values are calculated with voltage supplied by the device, and the current measured in meridians is expressed on a scale of 0–100. The procedure is performed by a well-trained technician in a quiet room with a temperature of 26–28 °C and constant humidity. Before the procedure, the participants first lie in a supine position for a 15-min rest. The 24 modified Yuan points are measured one by one, starting from the left side and following the order of Lung (LU9), Pericardium (PC7), Heart (HT7), Small Intestine (SI4), Triple Heater (TH4), Large Intestine (LI5), Spleen–Pancreas (SP3), Liver (LR3), Kidney (KI3), Urinary Bladder (BL65), Gallbladder (GB40), and Stomach (ST42). The TCM pattern and MEAD measurement are assessed before and after adjuvant chemotherapy.
3. Side effects of adjuvant chemotherapy such as nausea, vomiting, anorexia, mucositis, diarrhea, fatigue, hand–foot syndrome, paresthesia, neutropenia, anemia, and thrombocytopenia are graded using the Common Terminology Criteria for Adverse Events (CTCAE) scale, a commonly used tool [19]. These 111 items are selected from a longer list due to their incidence in this group of breast cancer and their considerable subjective and objective components, and because they usually partially or completely resolve by the time of the next cycle of chemotherapy [20]. Patients’ symptoms are recorded and graded from 1 to 5 to indicate if, after each cycle of chemotherapy administration, the side effect has occurred. For example, grade 2 nausea means “oral intake decreased without significant weight loss, dehydration or malnutrition.”

### Safety assessments

In addition to the focus on prognosis and QOL, TCM pattern, meridian energy, and chemotherapy-related side effects of breast cancer patients with CHM, the safety of the CHMs taken during the period of adjuvant chemotherapy is also a major focus. Routine blood tests and liver and kidney function tests are registered at baseline, and the occurrence of any functional damage after taking CHM is evaluated at each follow-up visit. Moreover, tumor markers (carcinoembryonic antigen [CEA] and cancer antigen [CA15-3]) and routine image examinations (computed tomography and breast ultrasonography) are recorded to estimate whether an increased chance for tumor progression occurs in the CHM group. In addition, based on the CTCAE issued by the National Cancer Institute (NCI), all AEs including toxicity and side effects must be reported throughout the course of the study. In case of any serious AE occurring, experimental intervention should be ceased immediately and proper treatments must be provided. At the same time, the severity of the AE and the relationship with CHM should be evaluated.

### Sample size calculation

The primary objective of the research is the 3-year DFS rate, which we used as the basis for the calculation of sample size. According to a relevant large-scale cohort study [21], the 3-year DFS rate of patients with early breast cancer after surgery is about 85%, and we expect it to increase to 95% with CHM treatment. The sample size for differences between two independent proportions was calculated by a G-Power (V.3.1) formula to ensure 80% power using a two-sided test with a significance level of α = 0.05. We need 141 participants in each cohort. Considering a projected dropout rate of 10%, the sample size of each cohort should be 155 and the total sample size should be 310.

### Statistical analysis

All statistical analyses will be performed in SPSS version 17.0 (SPSS Inc., Chicago, IL, USA). Statistical significance will be defined as *P* < 0.05. Based on the data distribution, the measurement data will be analyzed by *t* test or the Mann–Whitney *U* test, and the counting data by the χ^2^ test or Fisher’s exact test. Kaplan–Meier survival analyses will be used with regard to the DFS, and the log-rank test will be used between the two cohort groups. Repeated measures analysis of variance (ANOVA) and linear mixed models will be used to evaluate the repeated measures such as QOL, TCM pattern, and MEAD related to breast cancer and to compare the differences between the two groups. Therapeutic efficiency will be analyzed using the data of the full analysis set (FAS) and per protocol set (PPS); safety evaluation will be based on the data of the safety analysis set (SS) for statistical analysis.

## Discussion

The promising effects of TCM usage in the treatment of breast cancer have been progressively demonstrated. Previous studies have reported reduced patient mortality in those receiving combined TCM as compared to non-TCM users [22, 23]. A previous study found that CHM is helpful in both increasing tumor response during chemotherapy and improving QOL, and that it also has a partial effect in prolonging survival in patients with breast cancer [24]. Herb–drug interactions are the critical concern of clinicians, because the concomitant CHM may impact the efficacy of chemotherapy, which may lead to lost opportunities to overcome the disease [25]. Therefore, more powerful evidence is still needed to evaluate the association between CHM and the outcome of cancer treatment. We have presented the design of a prospective observational cohort study to evaluate the prognosis of adjuvant chemotherapy using CHM followed for up to 3 years for curatively early stage breast cancer. This study can provide objective evidence of TCM intervention in breast cancer treatment and also provide a reference on syndrome differentiation for TCM practitioners.

TCM theory and practice dictate that a positive therapeutic response is dependent on proper syndrome differentiation. The pattern type is caused by different states of yin-yang and *qi*-blood, which are dynamic and are influenced by different disease courses. However, very few studies have described changes in TCM patterns during chemotherapy in cancer patients. Only one longitudinal study by Huang et al. speculated that patients with breast cancer continued to have yang deficiency through observing low meridian energy during a 3-month period of chemotherapy [26]. The relationship between TCM pattern type and meridian energy still needs to be clarified. Through this study, we will also understand the changes in the syndromes of patients before and after adjuvant chemotherapy to provide TCM clinicians with therapeutic guidelines. Meanwhile, it is possible to investigate whether there is an impact on the TCM pattern type and meridian energy after CHM intervention. This will improve not only the accuracy of syndrome differentiation but also the pertinence of CHM.

Some limitations of this study are as follows. First, the study is an observational study, so the evidence level is lower than that of an RCT. TCM treatments are individualistic and dynamic in approach because they are based on clinical signs and symptoms at different stages, so there may not be a standard treatment for a population [27]. Moreover, the difficulties of randomization and blinding, selection of controls, and patient’s willingness and motivation to complete a study are other issues in conducting an RCT [28]. Although incorporating TCM principles in an RCT poses a challenge, we still can employ an evidence-based model for this traditional practice [29]. Second, baseline characteristics between the two cohorts may be imbalanced if the patients tend to choose combined CHM treatment in stage I. To resolve this problem, we will analyze the data by propensity score matching to control for some possible related factors. Third, the evidence supporting our TCM Treatment for Breast Cancer protocol is not sufficient. The protocol was developed in 2016, and it has not since been popularized due to a lack of large-scale clinical research. This will influence the rationality of treatment using this protocol, and our results may not be representative of the whole population. Finally, queue migration and the long time required for follow-up are both challenges in a cohort study. One way to avoid such problems is to strengthen the role of the cancer case manager in effective follow-up of the participants.

This proposal outlines the methodology for an observational study of adjuvant chemotherapy combined with CHM on the prognosis of patients with early breast cancer; the study adheres to the Standard Protocol Items: Recommendations for Interventional Trials (SPIRIT) statement [30]. The SPIRIT checklist is provided as Additional file 1. Results of this study should help provide support and parameters for the use of integrative TCM and anticancer treatments in patients with breast cancer.

## Supplementary information

**Additional file 1.** SPIRIT 2013 checklist: recommended items to address in a clinical trial protocol and related documents.

## Data Availability

The datasets collected and/or analyzed during the current study are available from the corresponding author upon request.

## Abbreviations

AE: Adverse event
BCQ: Body Constitution Questionnaire
CHM: Chinese herbal medicine
CTCAE: Common Terminology Criteria for Adverse Events (version 4)
DFS: Disease-free survival
EBCTCG: Early Breast Cancer Trialists’ Collaborative Group
ECOG: Eastern Cooperative Oncology Group
FACT-B: Functional Assessment of Cancer Therapy - Breast Cancer
MEAD: Meridian Energy Analysis Device
NCCN: National Comprehensive Cancer Network
NCI: National Cancer Institute
QOL: Quality of life
RCT: Randomized controlled trial
TCM: Traditional Chinese medicine
